# Association of Plasma Bilirubin Levels With Peripheral Arterial Disease in Chinese Hypertensive Patients: New Insight on Sex Differences

**DOI:** 10.3389/fphys.2022.867418

**Published:** 2022-04-14

**Authors:** Yumeng Shi, Wei Zhou, Mingshu Cheng, Chao Yu, Tao Wang, Lingjuan Zhu, Huihui Bao, Lihua Hu, Ping Li, Xiaoshu Cheng

**Affiliations:** ^1^ Department of Cardiovascular Medicine, The Second Affiliated Hospital of Nanchang University, Nanchang, China; ^2^ Jiangxi Provincial Cardiovascular Disease Clinical Medical Research Center, Nanchang, China; ^3^ Center for Prevention and Treatment of Cardiovascular Diseases, The Second Affiliated Hospital of Nanchang University, Nanchang, China; ^4^ China Jiangxi Wuyuan County Fuchun Hospitals, Shangrao, China; ^5^ Department of Cardiovascular Medicine, Peking University First Hospital, Beijing, China

**Keywords:** total bilirubin, ankle-brachial index, peripheral arterial disease, hypertension, U-shaped curve, males

## Abstract

**Background and aims:** Previous studies have indicated that Plasma total bilirubin (TBiL) might play an essential role in peripheral arterial disease (PAD). However, the effects of different levels of TBiL on PAD development remain uncertain. We aimed to examine the TBiL and the prevalence of PAD among Chinese adults with hypertension, with particular attention paid to sex differences**.**

**Methods:** A total of 10,900 hypertensive subjects were included in the current study. The mean age of our study participants was 63.86 ± 9.25 years, and there were 5,129 males and 5,771 females. The outcome was peripheral arterial disease (PAD), defined as present when the ankle-brachial index (ABI) of either side was ≤0.90. The association between TBiL and PAD was examined using multivariate logistic regression analysis and the restricted cubic spline.

**Results:** Of 10,900 hypertensive participants, 350 (3.21%) had PAD, and the mean plasma total bilirubin was 14.66 (6.86) μmol/L. The mean TBiL was 15.67 μmol/L in men and 13.76 μmol/L in women. The smoothing curve showed that a U-shaped curve association existed between TBiL and the prevalence of PAD in Chinese adults with hypertension. When stratified by sex, TBiL was significantly U-shaped associated with PAD among men but not women. Among males, the inflection point was 11.48 μmol/L; to the left inflection point, the effect size and 95% CI were 0.08, 0.01, 0.66, respectively; to the right inflection point, OR, 5.16; 95% CI,1.64, 16.25.

**Conclusions:** We found an independent U-shaped association between TBiL and the prevalence of PAD among hypertensive subjects and a differential association between men and women. We further revealed a turning point by threshold effect analysis.

## Introduction

As a prevalent cardiovascular disease (CVD), peripheral arterial disease (PAD) has a high fatality rate (Gerhard-Herman et al., 2017), and in recent years, the incidence rate of PAD has increased year by year (Eraso et al., 2014). Hypertension is one of the significant risk factors for PAD; the number of patients with hypertension is estimated at 245 million in China (Wang et al., 2018). Several studies have shown that increases in blood pressure are strictly related to increased PAD risk (Kannel and McGee, 1985; Murabito et al., 2002; Diehm et al., 2004). Therefore, there is an urgent need to identify novel modifiable risk factors to inform PAD prevention in the hypertensive population. The ankle-brachial index (ABI) is a noninvasive method for the diagnosis and detection of PAD and is widely used for risk assessment of atherosclerosis and other cardiovascular diseases (Schröder et al., 2006), and PAD was defined as an ABI of ≤0.90 in either leg (Aboyans et al., 2018).

The effects of serum total bilirubin (TBiL) on CVD risk have received considerable attention (Breimer et al., 1995; Lin et al., 2006; Huang et al., 2016; Lapenna et al., 2018). Bilirubin is a potent antioxidant under physiological conditions; a higher TBiL level could be a protective factor for atherosclerosis (Stocker et al., 1987). At the same time, some studies have shown that elevated TBiL levels were linear negatively correlated with coronary artery disease (CAD) (Schwertner et al., 1994; Song et al., 2014; Akboga et al., 2015), arterial stiffness (Huang et al., 2016), and PAD (Perlstein et al., 2008; Ozeki et al., 2018; Lan et al., 2020). Excessive TBiL is probably an indicator of the potential liver cell damage associated with an increased risk of CVD (Kunutsor et al., 2014). Nevertheless, the possible effect of excessive TBiL on the risk of increased PAD has not been examined in previous studies.

As the above studies did not discuss the nonlinear relationship between TBiL and PAD, and there were limited data in hypertensive participants, the present study aimed to assess the genuine dose-effect relationship between the TBiL and the prevalence of PAD in Chinese hypertensive subjects to address this gap in knowledge, as mentioned earlier.

## Methods

### Study Design and Population

All patients gave written informed consent. The Ethics Committees of the Institute of Biomedicine, Anhui Medical University, and the Second Affiliated Hospital of Nanchang University approved the study protocols. In the current study, we included rural subjects from the ongoing China H-type hypertension Registry Study (Registration number: ChiCTR1800017274). The China H-type hypertension Registry Study is a real-world observational study conducted in Wuyuan, Jiangxi Province, China, that began in July 2018. This study aimed to establish a national registry of patients with hypertension, investigate the prevalence and treatment of hypertension in China, and assess the related factors affecting its prognosis. Details regarding the inclusion and exclusion criteria of the study have been described elsewhere (Li et al., 2021; Shi et al., 2021). Eligible participants were men and women aged 18 years and older diagnosed with hypertension. The exclusion criteria of the study included1.psychological or nervous system impairment resulting in an inability to demonstrate informed consent, 2.unable to be followed-up according to the study protocol or plans to relocate soon, and 3.patients who are not suitable for inclusion or long-term follow-up as assessed by the study physicians.

A total of 14,234 participants met the inclusion and exclusion criteria. Subjects were excluded if they had missing ABI (*n* = 3328) and TBiL values (*n* = 6). The final analysis included 10,900 participants (Supplementary Figure S1).

### Data Collection

Baseline information on sociodemographic characteristics, lifestyle habits, comorbidities, and medication use was obtained through in-person interviews conducted by trained researchers according to a standard operating procedure. Anthropometric parameter indicators, including weight, height, waist circumference (WC), systolic blood pressure (SBP), diastolic blood pressure (DBP), and ABI measurements, were collected. BMI was calculated as the body weight in kilograms divided by the square of the height in meters (kg/m^2^).

All the study subjects were told one day in advance that fasting venous blood samples would be collected the next morning. After an overnight fast of 12–15 h, blood samples were collected utilizing venipuncture and were immediately frozen and stored at −80°C until analysis. The measured variables included total bilirubin (TBiL), serum homocysteine (Hcy), serum creatinine, lipids, and fasting plasma glucose (FPG). The formula used for the estimated glomerular filtration rate (eGFR) was the Chronic Kidney Disease Epidemiology Collaboration (CKD-EPI) equation (Levey et al., 2009). These parameters were measured using automatic clinical analyzers (Beckman Coulter) at the Biaojia Biotechnology Laboratory, Shenzhen, China.

### Ankle-Brachial Index Measurement

The ankle-brachial index (ABI) was automatically measured with the subject in the supine position after resting for more than 10 min using an Omron Colin BP-203RPE III device (Omron Health Care, Kyoto, Japan) and calculated for each leg by dividing the SBP obtained at the ankle level in the respective leg by the SBP of the brachial artery (Aboyans et al., 2012). The lowest value of the ABI was used in the analysis. PAD was defined as an ABI of ≤0.90 in either leg (Aboyans et al., 2018).

### Other Definitions

Hypertension was defined as SBP ≥140 mm Hg and/or DBP ≥90 mm Hg or if the individual was on antihypertensive medication in the past two weeks (Unger et al., 2020). Diabetes mellitus was defined as a self-reported physician diagnosis of diabetes or FPG concentration of ≥7.0 mmol/L or the use of glucose-lowering drugs.

### Statistical Analysis

We divided the study population into three groups based on Tertiles of TBiL levels for each gender. Baseline characteristics are presented as the mean ± SD for continuous variables and the count (percentage) for categorical variables. Differences in population characteristics were compared using one-way Analysis of Variance (ANOVA) test or chi-square test. Plasma concentrations of TBiL were expressed as μmol/L. The distribution of plasma concentrations of TBiL was strongly skewed toward the left. Thus, we performed the Log10 transformation (LgTBiL) before analysis. Multivariate logistic regression was used to investigate the association between LgTBiL and the prevalence of PAD. We constructed three models: Model 1 was not adjusted; Model 2 was adjusted for sex (only for overall population), age, BMI, SBP, DBP; and Model 3 was adjusted for sex (only for overall population), age, BMI, SBP, DBP; smoking status, drinking status, diabetes mellitus, stroke, coronary heart disease (CHD), homocysteine (Hcy), fasting plasma glucose (FPG), triglycerides (TG), low-density lipoprotein cholesterol (LDL-C), aspartate aminotransferase (AST), alanine aminotransferase (ALT), estimated glomerular filtration rate (eGFR), antihypertensive drugs, glucose-lowering drugs, lipid-lowering drugs. The regression analyses model selected the variables because of their clinical importance, statistical significance in the univariable analysis, and the effect of the potential confounder estimates individually changed by at least 10% (Greenland, 1989). According to published guidelines and studies, the main risk factors of PAD are smoking, hypertension, diabetes, abnormal lipid metabolism, obesity, and family history of cardiovascular disease (Eraso et al., 2014; Lu et al., 2014).To characterize the shape of the relationship between LgTBiL and PAD prevalence, a generalized additive model and smooth curve fitting (penalized spline method) were performed (Sullivan et al., 2015). If nonlinearity was detected, we first used a recursive algorithm to calculate the inflection points and then constructed a two-segment binary logistic model on both sides of the inflection points. As additional exploratory analyses, possible modifications of the LgTBiL effects on the prevalence of PAD in participants separated by the turning point of LgTBiL were also assessed for variables including age (<60 *vs*. ≥60 years), BMI (<24 *vs*. ≥24 kg/m^2^), current smoking (no *vs*. yes), current drinking (no *vs*. yes), eGFR (˂ 60 *vs*. ≥60 ml/min/1.73 m^2^), and diabetes mellitus (no *vs*. yes). A 2-tailed *p* < 0.05 was statistically significant in all analyses. Empower (R; www.empowerstats.com; X&Y Solutions, Inc., Boston, MA, United States) and the statistical package R (http://www.R-project.org, The R Foundation) were used for all data analyses.

## Results

### Study Participants and Baseline Characteristics

As shown in the flow chart (Supplementary Figure S1), a total of 10,900 hypertensive subjects were included in the current study. The mean age of our study participants was 63.86 ± 9.25 years. There are 5,129 males and 5,771 females.

The baseline characteristics of the study participants stratified by TBiL Tertiles of gender are summarized in Table 1. Among men, mean (SD) age was 63.92 (9.62) years, the mean TBiL was 15.67 μmol/L; the number of patients with PAD was 194 (3.78%). Among women, mean (SD) age was 63.80 (8.91) years, and the mean TBiL was 13.76 μmol/L. The number of patients with PAD was 156 (2.70%). Among males, the population with higher TBiL levels had higher values for BMI, DBP alcohol consumption, FPG, AST, ALT, eGFR, and diabetes mellitus, and lower values for smoking (all *p* < 0.01). The female population with higher TBiL levels had higher values for BMI, Hcy, FPG, AST, ALT, eGFR, diabetes mellitus, and CHD, and lower values for smoking, TG, LDL-C, stroke (all *p* < 0.01). There were no statistically significant differences among all groups regarding age, SBP, Hcy, TG, LDL-C stroke, CHD, antihypertensive drugs, glucose-lowering drugs, or lipid-lowering drugs among males (all *p* > 0.05). Moreover, there was no significant difference among women in age, drinking, SBP, DBP, antihypertensive drugs, hypoglycemic drugs, or lipid-lowering among all groups (all *p* > 0.05).

**TABLE 1:** Baseline characteristics of study participants.

	Male				Female			
	TBiL (μmol/L)^a^ Tertiles				TBiL (μmol/L)^a^ Tertiles			
Characteristics^b^	Tertiles1	Tertile 2	Tertile 3	P value^c^	Tertiles1	Tertile 2	Tertile 3	*P* value^d^
TBiL range	<12	12-16.7	≥16.7	<0.001	˂10.07	10.07-14.90	≥14.90	<0.001
N	1697	1701	1731		1901	1920	1950	
Age,year	64.32 ± 9.19	63.92 ± 9.69	63.54 ± 9.94	0.059	63.87 ± 9.35	63.81 ± 8.75	63.72 ± 8.64	0.881
BMI,kg/m^2^	23.05 ± 3.54	23.41 ± 3.48	23.61 ± 4.83	<0.001	23.56 ± 3.50	23.83 ± 3.64	23.97 ± 3.69	0.002
Current smoking, N (%)	911 (53.68%)	867 (50.97%)	765 (44.19%)	<0.001	142 (7.47%)	95 (4.95%)	88 (4.51%)	<0.001
Current drinking, N (%)	591 (34.83%)	721 (42.41%)	820 (47.37%)	<0.001	106 (5.58%)	116 (6.04%)	115 (5.90%)	0.819
SBP, mmHg	147.02 ± 18.42	146.17 ± 17.45	146.70 ± 18.01	0.371	150.69 ± 18.45	150.08 ± 16.96	149.59 ± 16.95	0.146
DBP, mmHg	89.48 ± 11.16	90.14 ± 10.96	91.41 ± 10.98	<0.001	87.77 ± 10.76	87.72 ± 10.06	88.01 ± 10.10	0.658
Laboratory data								
Hcy,μmol/L	19.96 ± 12.60	20.45 ± 13.57	21.09 ± 14.65	0.051	15.35 ± 6.42	15.42 ± 6.61	16.52 ± 8.72	<0.001
FPG, mmol/L	5.96 ± 1.43	6.06 ± 1.50	6.18 ± 1.56	<0.001	6.17 ± 1.62	6.22 ± 1.63	6.38 ± 1.78	<0.001
TG, mmol/L	1.65 ± 1.42	1.64 ± 1.11	1.67 ± 1.22	0.647	1.96 ± 1.37	1.86 ± 1.16	1.86 ± 1.14	0.014
AST, U/L	26.27 ± 15.14	27.15 ± 11.98	30.46 ± 32.17	<0.001	23.63 ± 7.62	25.47 ± 9.42	27.96 ± 12.10	<0.001
ALT, U/L	20.84 ± 14.97	21.84 ± 15.19	24.02 ± 29.16	<0.001	16.91 ± 9.77	18.43 ± 11.19	21.12 ± 14.46	<0.001
LDL,mmol/L	2.84 ± 0.78	2.88 ± 0.78	2.85 ± 0.78	0.287	3.06 ± 0.77	3.15 ± 0.85	3.12 ± 0.82	0.005
eGFR, mL/min/1.73 m^2^	83.78 ± 23.02	86.31 ± 19.57	88.41 ± 18.29	<0.001	88.96 ± 22.12	91.40 ± 18.89	92.24 ± 18.84	<0.001
Comorbidities, N (%)								
Stroke	147 (8.66%)	123 (7.23%)	133 (7.68%)	0.285	124 (6.52%)	84 (4.38%)	97 (4.97%)	0.009
CHD	77 (4.54%)	97 (5.70%)	99 (5.72%)	0.212	82 (4.31%)	79 (4.11%)	119 (6.10%)	0.007
Diabetes mellitus^$^	252 (14.85%)	264 (15.52%)	312 (18.02%)	0.029	349 (18.36%)	366 (19.06%)	435 (22.31%)	0.005
Medication use, N (%)								
Antihypertensive drugs	1117 (65.82%)	1124 (66.08%)	1121 (64.76%)	0.083	1258 (66.18%)	1257 (65.50%)	1278 (65.54%)	0.885
Glucose-lowering drugs	80 (4.71%)	73 (4.29%)	79 (4.56%)	0.835	113 (5.94%)	109 (5.68%)	118 (6.05%)	0.879
Lipid-lowering drugs	54 (3.18%)	65 (3.82%)	58 (3.35%)	0.571	65 (3.42%)	72 (3.75%)	67 (3.44%)	0.822

a TBiL value was log10-transformed.

b Data are presented as number (%) or mean ± standard deviation.

c Comparisons among TBiL tertiles in participants with Male.

d Comparisons among TBiL tertiles in participants with Female.

Abbreviation: BMI, body mass index; SBP, systolic blood pressure; DBP, diastolic blood pressure; Hcy, homocysteine; FPG: fasting plasma glucose; TG, triglycerides; LDL-C, low-density lipoprotein cholesterol; TBiL, total bilirubin; ALT, alanine aminotransferase, AST, aspartate aminotransferase; eGFR, estimated glomerular filtration rate; CHD, coronary heart disease.

### Associations Between TBiL and PAD

A multivariate logistic regression model was performed to evaluate the associations between TBiL and the prevalence of PAD. The effect values (ORs) and 95% confidence intervals (CIs) for full adjustment are listed in Table 2. Every 1 unit increase in LgTBiL was associated with 23% increased odds of PAD (OR 1.23; 95% CI 0.63-2.39), but the results did not reach statistical significance. We also handled TBiL as tertiles and as a categorical variable for sensitivity analysis. Compared with participants in T1, T2 (11.3-15.8) were not significantly reduced (T2: OR 0.76, 95% CI0.57, 1.00), and T3 (≥15.8) were not significantly increased (T3: OR 1.18 (0.90, 1.54), 95% CI 0.90, 1.54). In addition, the P for trend was not significant, showing that the relationship between TBiL and the prevalence of PAD was nonlinear. A similar trend was observed in both male and female patients.

**TABLE 2:** ORs and 95% CI of PAD incidence according to TBiL levels (μmol/L).

TBiL^a^	Events (%)	PAD *OR* (95%CI), *p* Value		
		Model 1	Model 2	Model 3
Male				
Tertile 1 (<12)	71 (4.18%)	Ref	Ref	Ref
Tertile 2 (12-16.7)	50 (2.94%)	0.69 (0.48, 1.00)	0.72 (0.49, 1.04)	0.77 (0.52, 1.13)
Tertile 3 (≥16.7)	73 (4.22%)	1.01 (0.72, 1.41)	1.13 (0.80, 1.59)	1.34 (0.93, 1.93)
P for trend	—	0.949	0.515	0.124
TBiL tertiles as a continuous variable	194(3.78%)	0.74 (0.33, 1.68)	0.98 (0.42, 2.26)	1.58 (0.65, 3.86)
Female				
Tertile 1 (<10.07)	61 (3.21%)	Ref	Ref	Ref
Tertile 2 (10.07-14.9)	43 (2.24%)	0.69 (0.47, 1.03)	0.73 (0.49, 1.08)	0.83 (0.55, 1.26)
Tertile 3 (≥14.9)	52 (2.67%)	0.83 (0.57, 1.20)	0.90 (0.61, 1.32)	1.06 (0.71, 1.59)
P for trend	—	0.305	0.559	0.794
TBiL tertiles as a continuous variable	156(2.7%)	0.48 (0.18, 1.23)	0.56 (0.21, 1.48)	0.89 (0.33, 2.44)
All participants				
Tertile 1 (<11.3)	132 (3.65%)	Ref	Ref	Ref
Tertile 2 (11.3-15.8)	92 (2.52%)	0.68 (0.52, 0.89)	0.69 (0.52, 0.91)	0.76 (0.57, 1.00)
Tertile 3 (≥15.8)	126 (3.47%)	0.95 (0.74, 1.22)	0.99 (0.77, 1.28)	1.18 (0.90, 1.54)
P for trend	—	0.663	0.926	0.264
TBiL tertiles as a continuous variable	350 (3.21%)	0.72 (0.39, 1.33)	0.78 (0.41, 1.46)	1.23 (0.63, 2.39)

a TBiL value was log10-transformed.

Model 1, unadjusted; model 2, adjusted for sex (only for overall population), age, BMI, SBP, DBP; model 3, full adjusted model, adjusted for sex (only for overall population), age, BMI, SBP, DBP; smoking status, drinking status, diabetes mellitus, stroke, CHD, Hcy, FPG, TG, LDL-C, AST, ALT, eGFR, antihypertensive drugs, glucose-lowering drugs, lipid-lowering drugs. Ref, reference.

### Threshold Effect Analysis of LgTBiL on PAD

**FIGURE 1:**
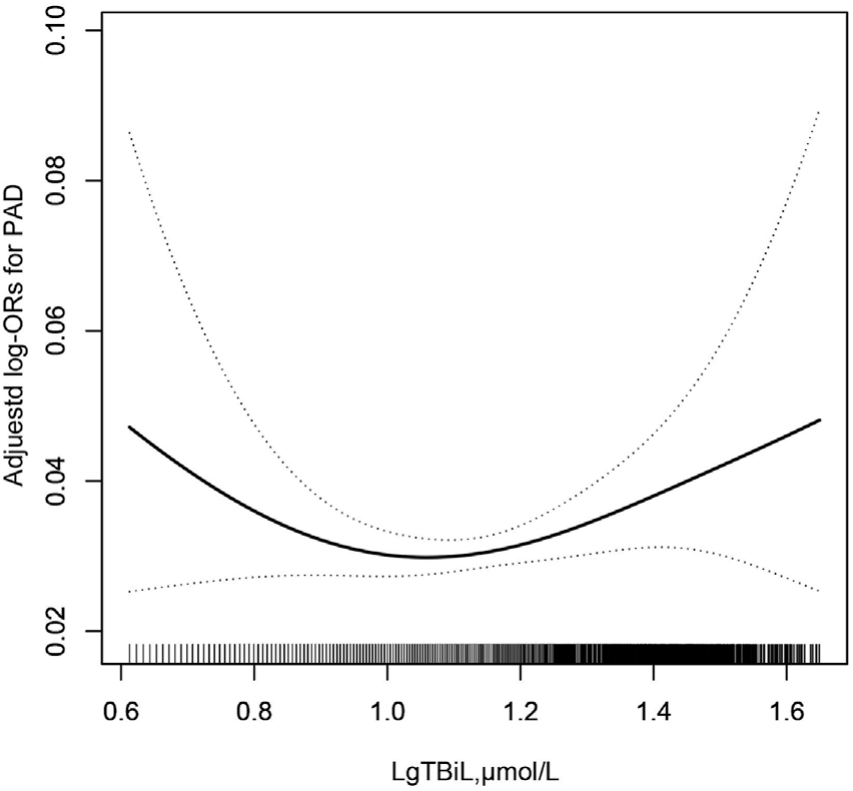
Association between LgTBil and the prevalence of PAD. A nonlinear association between TBil and the prevalence of PAD was found (*p* < 0.05). The solid line and dashed line represent the estimated values and their corresponding 95% confidence interval. Adjustment factors included sex, age, BMI, SBP, DBP; smoking status, drinking status, diabetes mellitus, stroke, CHD, Hcy, FPG, TG, LDL-C, AST, ALT, eGFR, antihypertensive drugs, glucose-lowering drugs, lipid-lowering drugs.

**FIGURE 2:**
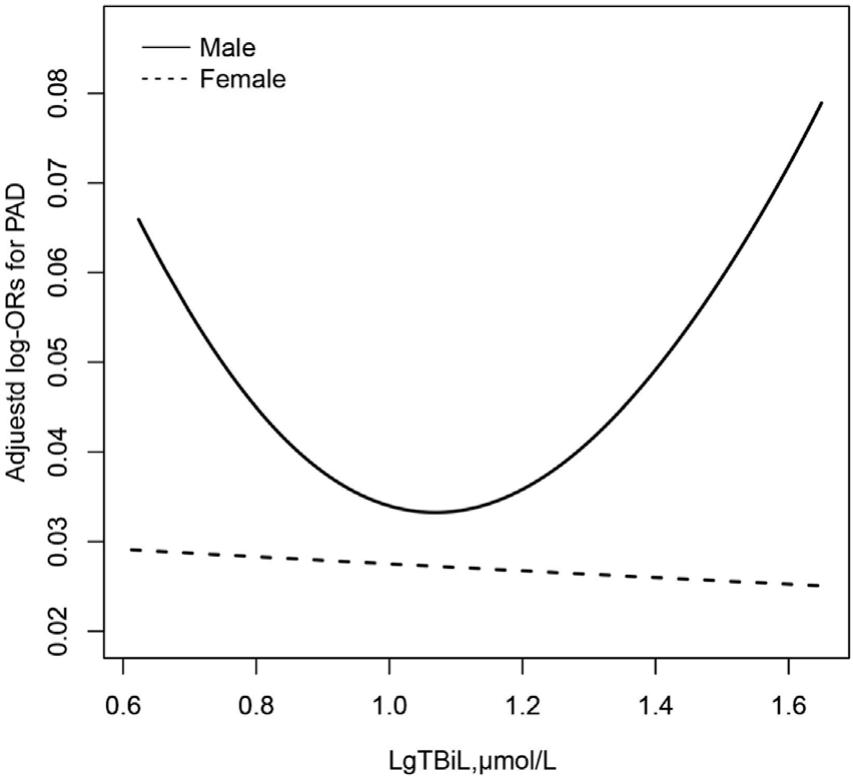
Association between LgTBil and the prevalence of PAD by sex. A nonlinear association between TBil and the prevalence of PAD by sex was found (*p* < 0.05). The solid line and dashed line represent the estimated values in male and female, respectively. The adjustment factors included age, BMI, SBP, DBP; smoking status, drinking status, diabetes mellitus, stroke, CHD, Hcy, FPG, TG, LDL-C, AST, ALT, eGFR, antihypertensive drugs, glucose-lowering drugs, lipid-lowering drugs.

To find the nonlinear relationship between TBiL and the prevalence of PAD, we used a generalized additive model and penalized spline method (Sullivan et al., 2015) (Figure 1). The smoothing curve showed that a U-shaped curve association existed between TBiL and the prevalence of PAD in Chinese adults with hypertension (after adjusting for sex, age, BMI, SBP, DBP; smoking status, drinking status, diabetes mellitus, stroke, CHD, Hcy, FPG, TG, LDL-C, AST, ALT, eGFR, antihypertensive drugs, glucose-lowering drugs, lipid-lowering drugs.). Given the differences in plasma TBiL levels between male and female participants (15.67 *vs*. 13.76 μmol/L), we further investigated the possible effect of sex on the TBiL-PAD association (Figure 2). The results supported a U-shaped curve association between TBiL and the prevalence of PAD in men but not women (Figure 2). We further fitted the relationship between TBiL and PAD using the two-piecewise logistic regression model (Table 3) and calculated that the inflection point was 11.48 μmol/L. Among the participants whose LgTBiL <11.48 μmol/L, there was a significant trend toward decreasing odds of PAD development with increasing LgTBiL (OR, 0.21; 95% CI: 0.05, 0.96). However, the odds of PAD development significantly increased with increasing levels of LgTBiL (OR,2.98; 95% CI:1.17, 7.62) in participants with LgTBiL ≥11.48 μmol/L. A similar trend was observed in male patients. Among the male, the inflection point was 11.48 μmol/L; on the left inflection point, the effect size and 95% CI were 0.08, 0.01, 0.66, respectively; on the right inflection point, OR, 5.16; 95% CI, 1.64, 16.25. Moreover, the log-likelihood ratio test was 0.005. However, TBiL is not associated with PAD in women.

**TABLE 3:** Results of two-piecewise logistic-regression model.

TBiL^a^	Male	Female	All Participants
Continuous	1.58 (0.65, 3.86) *p* = 0.311	0.89 (0.33, 2.44) *p* = 0.821	1.23 (0.63, 2.39) *p* = 0.539
Inflection point (K)	11.48	19.95	11.48
≤K Effect size OR (95% CI)	0.08 (0.01, 0.66) *p* = 0.020	1.38 (0.41, 4.61) *p* = 0.603	0.21 (0.05, 0.96) *p* = 0.044
>K Effect size OR (95% CI)	5.16 (1.64, 16.25) *p* = 0.005	0.03 (0.00, 8.37) *p* = 0.215	2.98 (1.17, 7.62) *p* = 0.023
*P* for log likelihood ratio test	0.005	0.175	0.015

a TBiL value was log10-transformed.

Two-piecewise logistic-regression model was used to calculate the threshold effect of the TBiL index. If the log likelihood ratio test >0.05, it means the two-piecewise logistic regression model is not superior to the single-line logistic regression model.

Adjusted for sex (only for overall population), age, BMI, SBP, DBP; smoking status, drinking status, diabetes mellitus, stroke, CHD, Hcy, FPG, TG, LDL-C, AST, ALT, eGFR, antihypertensive drugs, glucose-lowering drugs, lipid-lowering drugs.

### Subgroup Analyses

We performed exploratory subgroup analyses to assess the association between LgTBiL and the prevalence of PAD in two groups of participants separated by the turning point of TBiL (11.48 μmol/L) (Supplementary Figure S2). The effect of LgTBiL on PAD showed no significant difference in the following subgroups: age (<60 *vs*. ≥60 years), BMI (<24 *vs*. ≥24 kg/m^2^), current smoking (no *vs*. yes), current drinking (no *vs.* yes), eGFR (˂ 60 *vs*. ≥60 ml/min/1.73 m^2^), and diabetes mellitus (no *vs*. yes) in both groups (all P for interactions >0.05) after adjustment for sex, age, BMI, SBP, DBP; smoking status, drinking status, diabetes mellitus, stroke, CHD, Hcy, FPG, TG, LDL-C, AST, ALT, eGFR, antihypertensive drugs, glucose-lowering drugs, lipid-lowering drugs except for the stratifying variable. Similarly, we also found similar results in male subgroup analysis (Supplementary Figure S3).

## Discussion

For the first time, we found an independent U-shaped association between TBiL and the prevalence of PAD among men, we further revealed a turning point by threshold effect analysis. In contrast, no such relationship existed between TBiL and PAD for the female hypertensive patients.

Several previous studies have examined the relationships between TBiL levels and PAD (Perlstein et al., 2008; Ozeki et al., 2018; Lan et al., 2020). Ozeki et al. (2018)reported that serum bilirubin concentration was significantly negatively associated with PAD prevalence in 935 cardiology patients. Lan et al. (2020)conducted a cross-sectional study that included 543 participants with hypertension (mean age: 62.7 ± 12.4 years). The results showed that every 1 unit increment of TBiL was associated with an 8.6% (OR, 0.914; 95% CI: 0.845-0.990) lower risk of PAD; in addition, an independently negative relationship between TBiL and PAD (OR, 0.884; 95% CI: 0.792-0.985) was found in males but not in females. A cross-sectional examination from the National Health and Nutrition Examination Survey (1999–2004) analyzed 7075 adults with available on the ankle-brachial index, serum total bilirubin level, and PAD risk factors. The results showed that a 0.1 mg/dl increase in bilirubin level was associated with a 6% reduction in the odds of PAD (OR 0.94 [95% CI 0.90 to 0.98]), and this association is more influential in men than in women (Perlstein et al., 2008). However, these studies did not discuss a nonlinear relationship between TBiL and PAD.

Some new insights were demonstrated in hypertensive patients in the current study. Our in-depth study showed that the association between TBiL and PAD prevalence was not a simple linear association but a U-shaped curve, suggesting that low and high TBiL levels were associated with increased PAD prevalence. The reasons for these contradictory findings might be the different total serum bilirubin levels, and the distribution of total serum bilirubin levels may vary depending on gender (Walden et al., 1986), race (Oda and Kawai, 2012; Jung et al., 2014), age (Boland et al., 2014), health status (Chen et al., 2011; Song et al., 2014), and sample size of the subjects. We conducted a cross-sectional study including 10,900 Chinese hypertensive subjects. The mean age of our study participants was 63.92 ± 9.25 years, and the mean serum total bilirubin was 14.66 ± 6.86 μmol/L. However, Ozeki et al.’s study (Ozeki et al., 2018) enrolled 935 Japanese cardiology patients (median serum bilirubin: approximately 8.55 μmol/L), and Lan et al.’s study (Lan et al., 2020) analyzed 543 Chinese participants with hypertension. The mean serum bilirubin was 12.2 ± 5.6 μmol/L. At the same time, Perlstein et al. (Perlstein et al., 2008)conducted a cross-sectional examination that included 7075 adults of various races; the median total bilirubin level was 11.97 (interquartile range: 8.55–13.68) μmol/L. Due to the small sample size and the relatively lower bilirubin levels of the above study, we speculate that the negative relationship might be part of the U-shaped curve in this study. Second, previous studies were carried out in patients with cardiovascular disease and hypertension and the general population, while the current study was conducted in participants with hypertension accompanied by hyperhomocysteinemia (HHcy). HHcy was defined as Hcy level ≥10 μmol/L (Qin and Huo, 2016). The mean serum Hcy was 20.50 (13.64) μmol/L and 15.77 (7.35) μmol/L among males and females, respectively. Recent studies have shown that hyperhomocysteinemia is associated with an increased risk of PAD (Liu et al., 2020). Hyperhomocysteinemia can directly or indirectly produce toxic effects on vascular endothelial cells by damaging vascular endothelial cells, promoting platelet activation, enhancing coagulation, producing cytotoxic reactive oxygen species, reducing antioxidation and proliferation of vascular smooth muscle cells, thus promoting atherosclerosis (Liu et al., 2020). According to previous studies, bilirubin is an antioxidant that can prevent atherosclerosis, but there is no study on the pathological mechanism between bilirubin and homocysteine. Therefore, the U-shaped correlation between bilirubin and PAD in this study can be guessed that the bilirubin level *in vivo* is not enough to resist the arteriosclerosis promoting effect of Hcy.

Lu et al. (Lu et al., 2014) conducted a meta-analysis of the association between cigarette smoking and PAD. The results demonstrated that smoking increased the risk of PAD. According to a national study of the prevalence and risk factors associated with peripheral arterial disease from China, the significant risk factors for PAD are smoking, hypertension, diabetes, abnormal lipid metabolism, obesity, etc. (Wang et al., 2019). Because the effect of these covariates on PAD is too significant to cover up the effect of serum bilirubin on PAD, the independent effect of serum bilirubin on PAD is only reflected after adjusting it.

To our knowledge, TBiL is a potent endogenous antioxidant protecting cells from a 10 000-fold higher concentration of oxidants (Breimer et al., 1994; Liu et al., 2006; Akboga et al., 2015); hence, lower bilirubin levels could induce oxidative stress and inflammation, which are related to the pathogenesis and development of arteriosclerosis (Leopold and Loscalzo, 2005). However, the exact mechanisms of excessive TBiL levels with PAD remain unknown. One possible reason that could account for the association between excessive TBiL and increased risk of PAD is that excessive TBiL, as during dominant, might indicate potential liver cell damage, such as hepatocellular or obstructive jaundice, which in turn causes elevated levels of transaminases and alkaline phosphatase (Franchini et al., 2010). The increased levels of transaminase and alkaline phosphatase are associated with an increased risk of CVD (Schindhelm et al., 2007; Kunutsor et al., 2014). Diseases of the liver may interfere with the production of the active metabolites of vitamin D(Lee et al., 2019), reduced serum vitamin D levels were associated with an increased risk of PAD (Yuan et al., 2019). Therefore, we suspect that excessive TBiL levels can not offset the decline of vitamin D levels on the prevalence of PAD.

The potential limitations of our study should also be noted. First, we cannot draw any causal relationship between serum bilirubin and PAD from the data because this is a cross-sectional study. Second, the serum bilirubin was only assessed at the baseline in the present study; multiple tests may make the results more accurate. Third, in our questionnaire, we did not collect the information of symptoms in common vascular presentations, which was our limitation. PAD was only defined by ABI in our study. Many studies used ABI alone for PAD diagnosis in epidemiology investigation. Nevertheless, it would be better if our study used Edinburgh Claudication Questionnaire to estimate intermittent claudication and CT angiography (CTA) to examine lower extremity (Yan et al., 2020). Lastly, this study was conducted on Chinese hypertension participants; the generalizability of the findings to other populations remains to be determined.

## Conclusion

In summary, this cross-sectional study showed a U-shaped curve for the prevalence of PAD with TBiL in hypertensive male patients, with a turning point at approximately 11.48 μmol/L. However, the above-mentioned U-shaped relationship is only observed in men but not in women. Therefore, we should regularly monitor the bilirubin level in clinical practice because it is too high or too low, increasing PAD incidence, especially in Chinese male hypertensive patients. Further well-designed prospective cohort studies are needed to determine the association causality and clarify the potential underlying mechanisms of TBiL in the prevalence of PAD.

## Data Availability

The raw data supporting the conclusion of this article will be made available by the authors, without undue reservation.
