# Supplementary material for: Association of Plasma Bilirubin Levels With Peripheral Arterial Disease in Chinese Hypertensive Patients: New Insight on Sex Differences
Source: Front Physiol. 2022 Apr 14;13:867418. doi: 10.3389/fphys.2022.867418 (PMC9047868; doi:10.3389/fphys.2022.867418)
Supplement: Supplementary file 1 [file Image1.pdf]

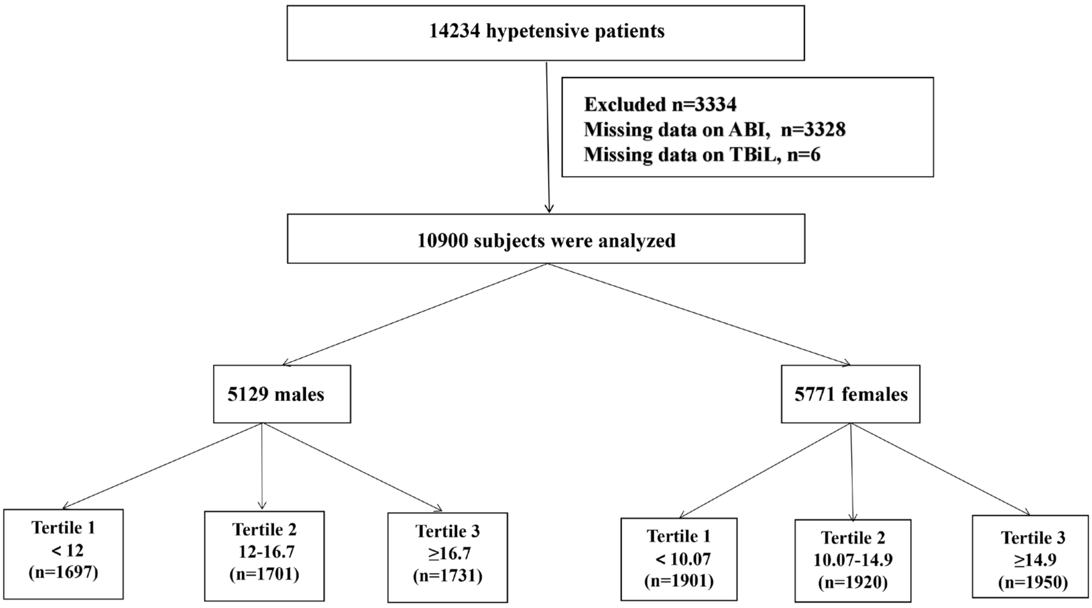

Figure S1 Flow chart of study participants

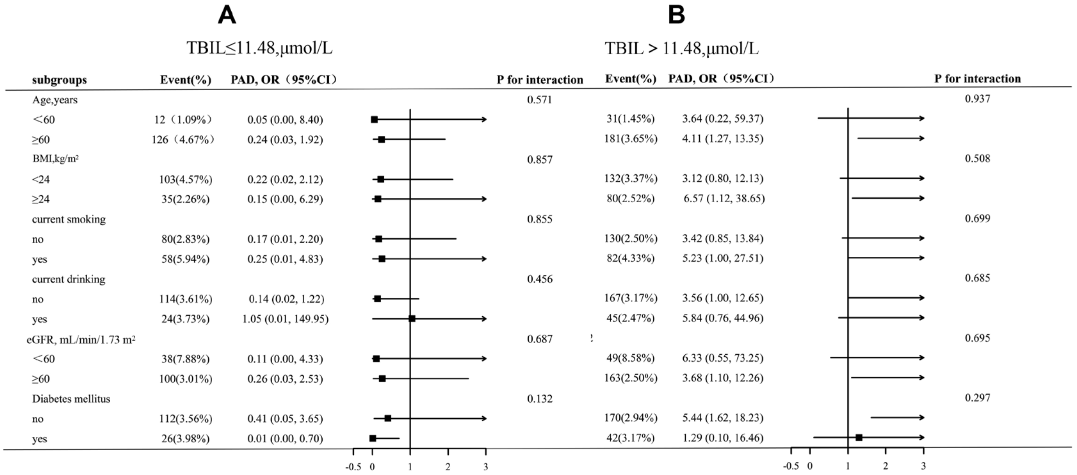

Figure S2 Stratified analysis for the PAD and LgTBil in various subgroups divided by 11.48 μmol/L. (A: TBil ≤11.48 μmol/lL, B: TBil >11.48 μmol/lL). *Each subgroup analysis adjusted for sex, age, BMI, SBP, DBP; smoking status, drinking status,diabetes mellitus, stroke, CHD, Hcy, FBG, TG, LDL-C, AST, ALT, eGFR, antihypertensive drugs, glucose-lowering drugs, lipid-lowering drugs， except for the stratifying variable.

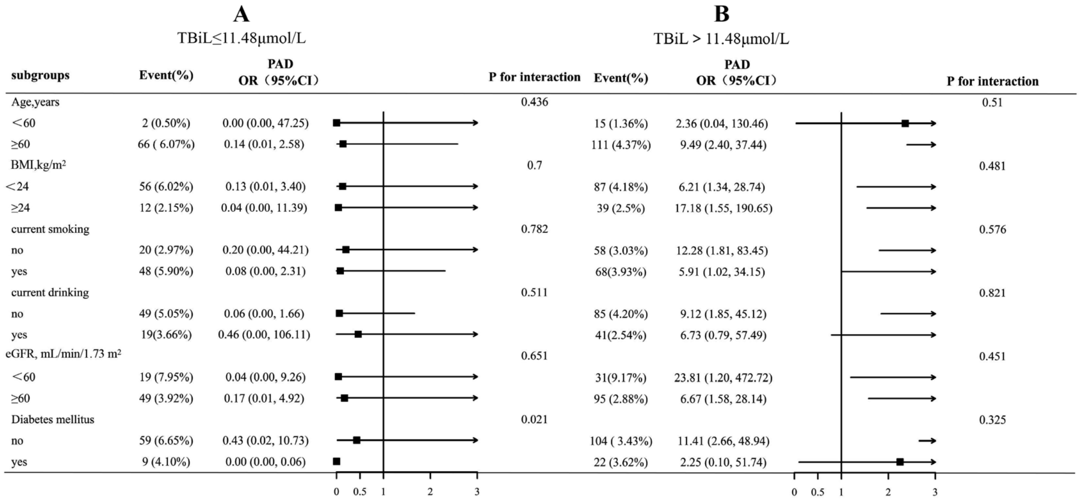

Figure S3 Stratified analysis for the PAD and LgTBil in various subgroups divided by 11.48 μmol/L among men. (A: TBil ≤11.48 μmol/lL, B: TBil >11.48 μmol/lL). *Each subgroup analysis adjusted for age, BMI, SBP, DBP; smoking status, drinking status,diabetes mellitus, stroke, CHD, Hcy, FBG, TG, LDL-C, AST, ALT, eGFR, antihypertensive drugs, glucose-lowering drugs, lipid-lowering drugs， except for the stratifying variable.
